# Evaluation of an intermediate minor oral surgery service in Plymouth, England and implications for future commissioning

**DOI:** 10.1038/s41405-025-00394-w

**Published:** 2026-01-04

**Authors:** Amirali Ziaebrahimi, Robert Witton

**Affiliations:** Peninsula Dental School, University of Plymouth, Plymouth, UK

**Keywords:** Health care, Oral conditions

## Abstract

**Objective:**

To evaluate a new primary-care based oral surgery service in Plymouth, England using Maxwells’ Dimensions of Quality framework.

**Methods:**

A retrospective observational study of referral, service and treatment outcome data.

**Results:**

Over a 35-month period the service received 1165 referrals, with 89% of patients referred receiving treatment within 63 days. Treatment provided was predominantly dento-alevolar surgery accounting for 82% of treatment provided, and soft tissue lesions accounting for 17%. The re-attendance rate for complications was 4%, and antibiotic use was 2%. Sedation was required for 17% of treatment cases. Cost analysis indicated a cost per case of between £138–196.

**Conclusion:**

The service meets the national clinical standards in oral surgery. High ongoing demand for referral of medium-complexity oral surgery cases from primary dental care to specialist providers/services is likely to continue. The opportunity to develop more primary care capacity has the potential to improve accessibility and convenience for patients and optimise use of NHS resources.

## Introduction

Oral surgery deals with the diagnosis and management of pathology of the mouth and jaws that requires surgical intervention. It involves the treatment of children, adolescents and adults, and the management of dentally anxious and medically complex patients [1]. The extraction of teeth and other minor oral surgery procedures are key disciplines in the practise of primary care dentistry, and ‘*surgical treatment*’ is part of the service expected from patients. There are occasions however, where the scope of care required is beyond the expertise and capability of a generalist practitioner in primary care [2]. In these circumstances patients are referred to a practitioner with enhanced skills (which may be a registered specialist in oral surgery or a non-specialist practitioner), and in a UK context this traditionally involved referral to an oral surgery department found in a hospital oral and maxillofacial department [3, 4]. Increasing volumes of oral surgery referrals to hospital units have been reported in the literature over the past twenty years, which has led to an increase in waiting times for patients, reduced access to care and overburden of hospital-based services [3, 4]. The primary care - specialist interface is a key organisational feature of many healthcare systems including the National Health Service (NHS) in the UK, and the increase in the number of referrals observed has wider consequences for the healthcare system particularly in times of increasing fiscal constraint in publicly funded dental services [5].

In response to these changes in practising behaviours, the NHS in England has developed commissioning guidance for oral surgery and has designated low to medium complexity cases, called Level 1 (e.g., extraction of erupted teeth, and uncomplicated third molars) as being commensurate with the level of experience expected of a dental practitioner in primary care [1]. Level 2 is designated for more complex cases which require enhanced skills and training in oral surgery (e.g., surgical removal of uncomplicated third molars requiring bone removal and soft tissue surgery) and Level 3 denotes complex oral surgery where there is an increased risk of complications, and therefore a registered specialist is required to perform the procedure and typically requires additional equipment and a hospital environment to manage the care safely (please refer to the clinical guideline for a comprehensive list of procedures in each level) [1].

National reviews and policy papers, including the 2011 *Review of Oral Surgery Services and Training* [6], 2013 *Securing Excellence in Commissioning NHS Dental Services* [7], and the 2014 *Five Year Forward View* [8], advocate for redesigning oral surgery pathways to improve cost-effectiveness, accessibility, and quality. These reforms aim to ensure that procedures historically managed in hospitals can be delivered safely and efficiently within community settings by suitably accredited and trained practitioners. In response to these policy drivers Level 2 (sometimes referred to as IMOS [intermediate oral surgery services]) have been developed in primary dental care across England over the past decade to reduce pressure on hospital referrals, improve accessibility, experience and convenience for patients and optimise use of NHS resources. Evaluation of services in Sheffield, Lancashire, and London have demonstrated clinical and cost-effectiveness with high levels of patient acceptability [9–11].

Plymouth is a small coastal city with an urban population of 265,000 [12], the surrounding area is predominantly rural, and the region is socio-economically deprived. The city and surrounding area are served by one hospital, where oral surgery services are provided alongside several IMOS operating within the city. There are approximately 150 primary dental care practices of varying sizes in the referral catchment  area. Despite the local availability of oral surgery services, high demand for oral surgery persists and a new IMOS was developed to support patient and practitioner need. To support high-quality service development an evaluation of the service was conducted according to Maxwell’s Dimensions of Quality – effectiveness, efficiency, equity, acceptability, accessibility, and apprioateness [13]. A range of frameworks exist for evaluating the quality of clinical services, we selected Maxwell’s framework as it emphasises the plurality of quality dimensions from a patient perspective. Furthermore, it has been used to evaluate previous oral surgery services allowing a degree of comparison to other studies.

## Description of the service

### Service location and accessibility

The IMOS is delivered from two sites in Plymouth city both located in high population density areas. One site in the southwest of the city, is in an area of high deprivation, the other is in the north of the city providing two access points to the service. Both sites provide free car parking, and both are accessible via frequent public transport links or by foot. Both sites are fully compliant with the Equality Act 2010, ensuring that services are inclusive and adaptable to the individual needs of patients. This includes step-free access, wheelchair accessibility, and support for patients with additional communication or mobility needs. Detailed accessibility information is publicly available via the providers website for each site. The service operates four days per week, between the hours of 9 and 5 aligned to its NHS contractual requirements. The IMOS is funded by the NHS to provide 6 sessions per week equating to 276 sessions over 46 weeks of the year, with an expectation that a minimum of 3-4 patients are treated per session including assessment and treatment.

### Clinical pathway

A description of the care pathway can be found in the national clinical standard including the procedures and conditions suitable for referral to a Level 2 (IMOS) [1]. The service provided is a referral-only model, integrated with NHS systems via a Referral Support Service (RSS) allowing general dental practitioners (GDPs) to refer patients that meet referral criteria according to the national clinical standard [1]. All referrals are screened by RSS for appropriateness before being distributed among providers. Should a referral be rejected as unsuitable for IMOS, it is returned to the RSS for appropriate re-direction.

The service is delivered by one registered specialist in oral surgery supported by oral surgery trained dental nurses with administrative support. The aim of the service is to complete treatment in two visits, with the first visit usually being a consultation to confirm the diagnosis and treatment plan. The service provides inhalational and intra-venous sedation according to patient need. Clinical activity is divided into consultation and treatment sessions. Consultations, either face-to-face or by phone are conducted in blocks of 6–8 per session. Treatment sessions include 3–4 local anaesthetic cases per list. On sedation days (1 day per week) two cases are planned for each session. A histo-pathology service is available alongside the service provided by a local hospital pathology service. All standard clinical governance reporting occurs alongside clinical activity data recording.

### Evaluation methods

The evaluation included a retrospective observational study of anonymized patient data including analysis of patient feedback. Reflections from the provider team delivering the service were also collected. A basic cost analysis of the service was also conducted.

### Retrospective data analysis

The retrospective analysis focused on patient demographics, attendance figures, number and type of surgical treatments provided and treatment outcomes.

### Data collection and analysis

Data were analysed for all IMOS cases accepted into the service between August 2022 and July 2025 (35 months). Postcodes of residence were used to determine in which Index of Multiple Deprivation (IMD) each patient treated in the service lived, records with an invalid postcode were excluded. Eighty-two patients in active care were excluded from the analysis due to incomplete care outcomes. Feedback from the provider team were collected via a short feedback interview and a basic cost analysis was undertaken based on the operating costs of the service (pay and non-pay costs) and an estimate for cost per case was derived.

All data were managed in accordance with local research governance and GDPR policies. Only anonymised data were handled, no personal identifiable data was shared with the study authors and all data extraction was conducted by the provider, always maintaining patient confidentiality.

## Results

### Referral data

In the reporting period, 1165 patients were referred to the service. Overall referral outcomes can be seen in Table 1.

**Table 1:** Referral outcomes Aug 2022 to July 2025.

Outcome	Total	% of Total Referrals
Treatment	1040	89
Failure to attend	45	4
Discharged due to repeated cancellation	1	<1
Patient cancelled	3	<1
Provider cancelled	1	<1
Treatment no longer required	20	2
Patient declined treatment	32	3
Not suitable for treatment in IMOS	23	2

**Fig. 1:**
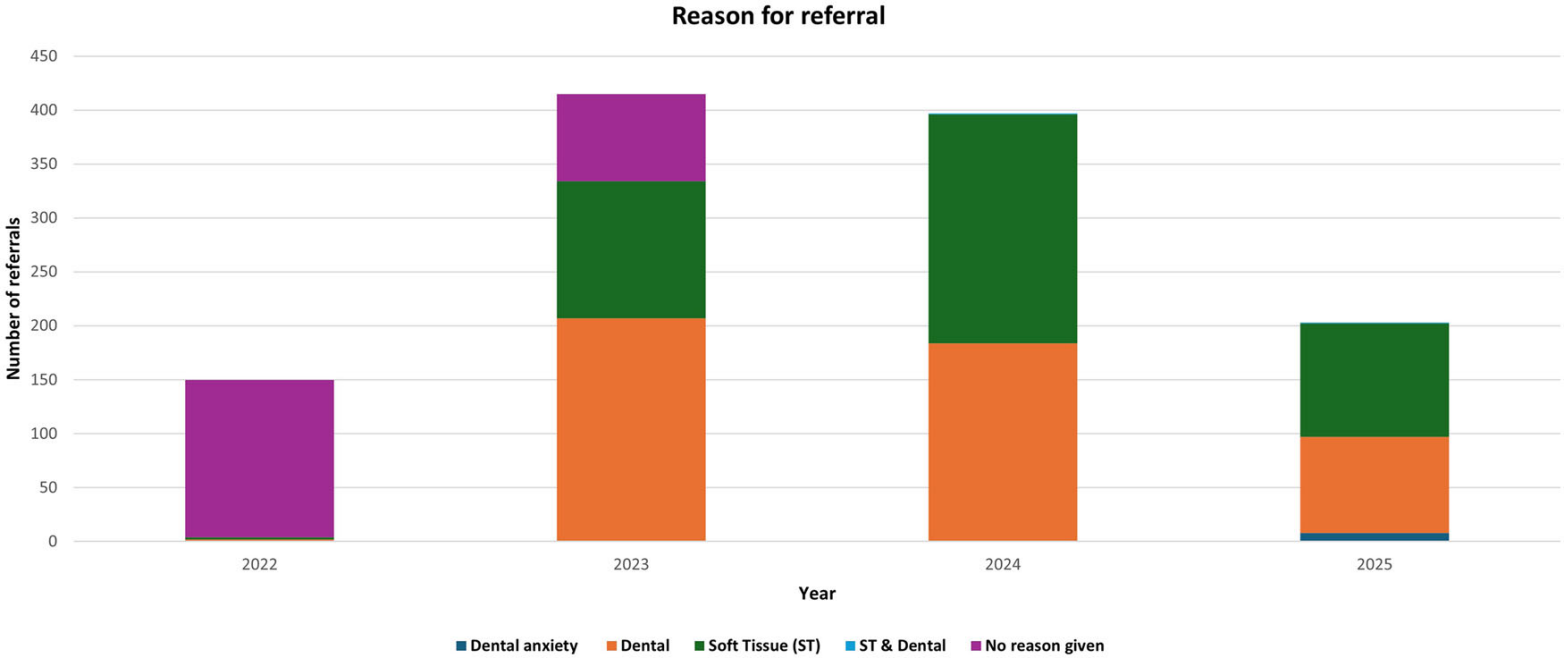
Reason for referral to the service August 2022 to July 2025. Reported across five categories. Purple = no reason given orange = dento-alveolar green = soft tissue blue = dento-alveolar & soft tissue and dark blue = dental anxiety.

The reason for referral can be seen in Fig. 1. Initially the service received referrals with no reason given, but this reduced over time as the service has matured. As the service has matured, it receives a similar number of referrals to manage soft and hard tissues.

In the evaluation period, the average time for initial contact with a patient after receiving a referral to the offer of an appointment was 4.3 days, referral to assessment was 26 days and assessment to treatment was 32.5 days.

### Patient characteristics

The average age of patients treated in the service was 48 years (age range 29 to 81 years). The split of males to females was 45% vs 55%. Data on ethnicity was not available for analysis nor their eligibility for free public dental care. The majority of referrals (88%) were from patients living in Plymouth city postcodes which is a travel distance of 5 miles or less. Analysis of postcode data revealed the majority of patients (55%) treated in the service (*n* = 572) were from IMD 1–4 representing areas which are more deprived. In comparison, only 24 patients (2%) had a postcode linked to IMD10, the least deprived area.

### Treatment provided

A full breakdown of the treatment provided for 1040 patients can be seen in Table 2.

**Table 2:** Treatment provided Aug 2022 to July 2025.

Treatment provided	Total	% of total treatments
Extraction	433	50
Surgical extraction	278	32
Apicectomy – molar	0	
Apicectomy – premolar	0	
Apicectomy – incisor or canine	2	<1
Frenectomy	2	<1
Alveoplasty	0	
Bone biopsy	0	
Soft tissue biopsy	149	17
Incision and drainage	1	<1
Closure of oro-antral communication	1	<1
Telephone consultation	278	
Post-op arrest of haemorrhage	1	
Post-op removal of sutures/dressing	2	
Post-op treatment of infected socket	43	
Prescription – antibiotics	23	
Prescription – other	23	
IV sedation required	143	
Inhalation sedation required	28	

Analysis of workload data indicated a number of appointments were used for additional radiography including CBCT, clinical photography and anxiety management. In addition, time in the service was given to; follow-up incomplete of referral information; liaison with general physicians to confirm medical status of referred patients and review appointments where clinically indicated. Overall, 7% of clinical time was lost due to the reasons specified in Table 1.

### Clinical quality indicators

**Fig. 2:**
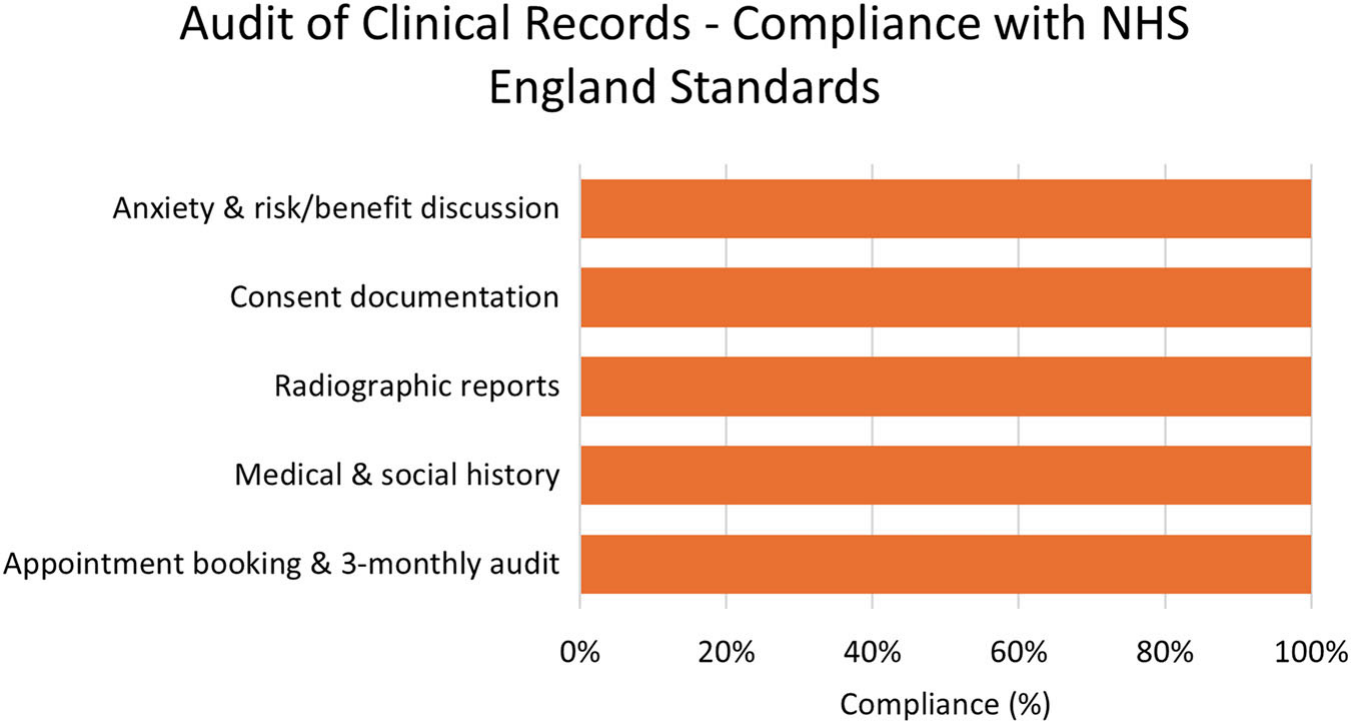
Clinical quality indicators August 2022 to July 2025. Compliance against five quality indicators specified in the NHS contract.

Clinical records are regularly audited each quarter as part of contractual monitoring of quality and to measure compliance with NHS clinical standards, the summary of these audits for the reporting period can be found in Fig. 2. The provider has a clinical incident reporting system and data were provided on two minor clinical incidents during the reporting period.

### Patient feedback

The service collects anonymised patient feedback in the form of Patient Reported Experience Measures (PREMs) and patients are contacted after treatment to share their feedback. 100% of patients (*N* = 114) responding felt involved in treatment decisions and reported excellent communication around risks, aftercare, and medication. No feedback expressed dissatisfaction or uncertainty regarding any aspect of care and 99% of patients said they were *extremely likely* or *likely* to recommend the service. No complaints have been received on the service.

### Provider feedback

Due to study limitations, formal qualitative study was not possible, so the clinical delivery team were asked to provide their reflections on the service via a short interview with the lead author. This was focused on challenges in setting up the service, and further improvements they think should be introduced. The main challenges reported by the provider team related to the integration of the service within the wider NHS and the administration of the care pathway, whereby referrals numbers were described as inconsistent, lacking key patient information or having poor quality radiographs.

One of the areas the provider felt significant improvements could be made was moving away from ‘paper-based referrals’ to the introduction of a digital referral system to enable fast, safe and secure transfer of patient referral information including radiographs. The provider team felt such a system would enable the service to treat more cases as they would spend less time on bureaucracy. The provider clinical team felt the use of a digital referral system would also improve data collection and improve efficiency in information exchange between the service, RSS, patient and referring practitioner.

### Cost analysis

A basic cost analysis was performed to examine the provider pay and non-pay costs in delivering the service, this identified the service was loss making, with a shortfall in funding compared to the actual costs of delivery of approximately 11%. Each session in the service attracts funding from the NHS at a rate of £437. The cost per case was sensitive to attendance rates and the number of visits required but ranged from £138 to £196 for a single episode of care.

## Key findings mapped to quality dimensions

### Effectiveness

The evaluation has demonstrated clinical effectiveness, with a 4% complication rate, only two patient safety incidents in the reporting period and robust compliance with clinical governance requirements and national NHS clinical standards.

### Efficiency

The service operates efficiently, with 89% of patients referred to the service receiving treatment. Referral data indicates almost all patients received treatment within 63 days (16 weeks) of referral by their primary care practitioner.

### Equity

Measuring equity is challenging as the service is referral only, and not all data related to patient characteristics was available for analysis. However, postcode analysis indicates most patients treated (55%) were from Plymouth city postcodes representing more deprived areas of the region (IMD 1-4).

### Acceptability

Feedback suggests high patient satisfaction, particularly around communication, pain management, and aftercare support. The service has received no complaints over the 35-month evaluation period.

### Accessibility

The service is delivered from two publicly accessible locations and offers a range of disability assistance improving accessibility. The service offers a range of appointments between 9-5 4 days per week.

### Appropriateness

The service appears to be meeting the need it was established to meet, very few referrals are rejected by the service (2%) indicating the appropriateness of referral criteria and pathway. The service has reduced demand on other local services by providing new capacity for almost 400 appointments per year.

## Discussion

This evaluation has demonstrated the new IMOS service is operating successfully and in accordance with national clinical standards in oral surgery [1]. Evidence indicates the service is efficient, clinically effective and has high patient acceptability and satisfaction. The main findings of this evaluation are similar to those of other published evaluations of primary-care based oral surgery services; however, this evaluation has several distinct differences, the first is its location in the Southwest which is different in context compared to areas where other evaluations have been conducted, most notably being a region with low delivery of primary care dentistry, it also included a more detailed analysis of quality which has not been a feature of other studies [9–11, 14]. Furthermore, the service appears to have a different treatment profile compared to other services, with a higher proportion of referrals to manage soft tissue lesions, which have increased as a proportion of total referral activity as the service has matured. For example, 9% of cases referred to a service in Lancashire [10] were for soft tissue biopsy, whereas this service is almost double at 17%. A 2018 study by Dyer and Lau [15] reported 23.4% of referrals were for oral medicine to a service in Yorkshire but this study pre-dates the latest NHS clinical standard in oral surgery [1]. The reasons for this higher proportion of soft tissue referrals are unknown, but it could relate to structural factors in the referral system, which favours referral of soft tissue lesions to this particular service because it has a histopathology service associated with it, rather than a higher incidence of oral-mucosal disease in the local population. This finding warrants further study, to determine the numbers and types of soft tissue lesions treated in the service, and the proportion of lesions giving cause for concern which require referral for suspected oral cancer, and if this observation is isolated to this service. As this would require a case note audit and use of person identifiable data, this was not possible in this study.

The need for additional behaviour management and use of sedation is also not commonly reported, in this service, 14% of patients treated had intra-venous sedation and 3% had inhalational. Antibiotic use was low and similar to other published evaluations [9, 14, 15] at 2% and complications rates were very low. Referral outcome data was similar to other published reviews with short waiting times and patients moving through the pathway efficiently, with the provider meeting NHS targets for timeliness of care.

Although the economic argument for locating more specialised oral surgery services in primary care is strong with significant benefits to the NHS, with gross savings estimated to be between £440 and 670 per case compared to a hospital [11, 16] the issue is more nuanced than economics alone as many hospital and dental hospitals have important roles in training undergraduate and postgraduate students in oral surgery and therefore require a range of cases with different complexities [11]. The cost per case, based on a single treatment visit is similar to other published estimates, however, the service overall is loss making for the provider with a shortfall in funding in the region of 11%. In addition, the basic cost-analysis has highlighted an apparent weakness in the service being small, compromising its financial sustainability due to disproportionate costs relative to contract size and revenue. This also impacts on the cost per case which is slightly higher than other published studies indicating this is an important issue to consider when commissioning such services. Due to these issues, the service also lacks resilience, with only sufficient funding to employ one oral surgeon thereby exposing the service to risks of disruption due to its sole practitioner model.

The lack of sustainable funding was a concern to the provider, and while the service is not sustainable, the provider explained they are able to sustain it because other services it delivers are profit making effectively subsiding the oral surgery service. Such issues need to be addressed, and realistic contracts offered by the NHS, which reflect the actual costs of delivering a service, if it wishes to expand the availability of specialised care in the community. This requires commissioners to work more collaboratively with providers to understand costs, so commissioning is more commercially appropriate and reflects dental market forces.

The providers main feedback on service improvement was linked to the above financial considerations and they felt strategic service expansion would improve resilience and cost-effectiveness in the service. Increased funding would allow more cases to be treated and employment of additional clinicians. Improved service sustainability would also provide opportunities for training and create opportunities for undergraduate and/or postgraduate students or local primary care practitioners to upskill in oral surgery, which could reduce referral demand long-term. Alongside care pathway improvements such as introduction of a digital referral system to improve the referral processes, these broader objectives were described by the provider as a lost opportunity to achieve better value and improved organisational outcomes from the service.

### Limitations

Like all evaluations of real-world services there were limitations in the data available, both data sent to the provider from RSS and data collected by the service, this prevented some analyses. For example, data on referral waiting times to other local oral surgery service providers was not available, and therefore, we were unable to measure the impact of the new service on local waiting times. Improving data collection and intelligence should be a key service improvement for all clinical services and agreement on a core national data set would enable standardised health impact and equity reporting. This work was undertaken by a year three undergraduate dental student participating in a dental public health summer studentship. As a result, there are limitations to the study, firstly due to funding and time constraints we were not able to conduct in depth qualitative interviews with stakeholders such as patients, staff delivering the service, referring practitioners and commissioners. This has limited the overall depth of the evaluation as we are not able to triangulate the findings or explore contextual factors that explain the quantitative data. Nonetheless, the data presented is valuable to add to the evidence base on IMOS effectiveness as other published evaluations were undertaken some time ago, pre-Covid or in parts of England where there are stark differences in dental market forces compared to the southwest [17].

Finally, the service is still new and developing, and 35 months represents a narrow window of evaluation, further evaluation is required as the number of patients treated in the service increases, service improvement initiatives are embedded, and national clinical guidance is refined.

## Conclusions

The provision of specialist dental services in primary care settings has been increasingly prioritised in UK health policy. In line with these national strategies, the evaluation of the IMOS service has indicated it meets the national clinical standards for level two oral surgery and is providing a high quality, clinical effective, accessible and acceptable service to patients accessing it according to Maxwell’s Dimensions of Quality. The rationale behind developing this service was to improve efficiency, reduce waiting times, and enhance patient experience, all while preserving clinical quality and safety. By relocating appropriate cases into a specialist-led primary care setting, the service has relieved pressure on local hospital-based oral surgery and increased capacity in primary care by handling almost 1200 low- and medium complexity cases that would otherwise need to be treated in existing services. Comparable initiatives across the UK have shown promising results, including shorter waiting times, cost savings, and high levels of patient satisfaction—particularly where care was delivered in familiar, community-based settings. This evaluation has additionally highlighted the risks of commissioning small-scale services, where there is a lack of service resilience and financial sustainability.

### Ethical approval

Completion of the Health Service Research Authority decision tool determined that ethical approval for the study was not required. The study is based on secondary analysis of anonymous routinely collected service data that is reported as part of service monitoring and performance. Consent was not required for retrospective data collection.

## Data Availability

All data used in the study were handled in accordance with local GDPR policies. The datasets used and/or analysed during the current study are available from the corresponding author on reasonable request.
