# Correction: Evaluation of an intermediate minor oral surgery service in Plymouth, England and implications for future commissioning

**DOI:** 10.1038/s41405-026-00401-8

**Published:** 2026-03-30

**Authors:** Amirali Ziaebrahimi, Robert Witton

**Affiliations:** Peninsula Dental School, University of Plymouth, Plymouth, UK

**Keywords:** Health care, Oral conditions

Correction to: *BDJ Open* 10.1038/s41405-025-00394-w, published online 04 January 2026

In the version of the article initially published, the Competing interests section was incomplete and has now been amended to “R.W. is an Editorial Board Member of BDJ Open. The other author(s) declare no competing interests”. This correction has been made to the HTML and PDF versions of the article.

